# Isolated adult hypoganglionosis presenting as sigmoid volvulus: a case report

**DOI:** 10.1186/1752-1947-5-445

**Published:** 2011-09-08

**Authors:** Irfan Qadir, Muhammad Musa Salick, Abrar Barakzai, Hasnain Zafar

**Affiliations:** 1 General Surgery Department, Aga Khan University Hospital, Stadium Road, Karachi 74800, Pakistan; 2 Histopathology Department, Aga Khan University Hospital, Stadium Road, Karachi 74800, Pakistan

## Abstract

**Introduction:**

Isolated hypoganglionosis is a rare cause of intestinal innervation defects. It is characterized by sparse and small myenteric ganglia, absent or low acetylcholinesterase activity in the lamina propria and hypertrophy of the muscularis mucosae, principally in the region of the colon and rectum. It accounts for 5% of all intestinal neuronal malformations. To the best of our knowledge, only 92 cases of isolated hypoganglionosis were reported from 1978 to 2009. Isolated hypoganglionosis usually manifests as enterocolitis or poor bowel function, and is diagnosed in infancy or childhood. We report the first case of isolated hypoganglionosis presenting with sigmoid volvulus in a 34-year-old woman.

**Case presentation:**

A 34-year-old Asian woman had progressively increasing abdominal pain and had not passed stool or flatus for two days. A physical examination revealed a distended abdomen with sluggish gut sounds. A computerized tomography (CT) scan demonstrated gross dilatation of the sigmoid colon (maximal diameter 14.3 cm) suggestive of sigmoid volvulus. During emergency laparotomy, sigmoidectomy with a side-to-side colorectal anastomosis was performed. Histopathology of the resected specimen showed occasional ganglion cells and hypertrophied nerve bundles in the muscle layers, suggesting hypoganglionosis. Colonoscopy was performed, and multiple full-thickness biopsies were taken that showed hypoganglionosis of the entire large bowel. Our patient underwent total colectomy with an ileorectal anastomosis. Subsequently our patient reported a dramatic improvement in her bowel function.

**Conclusions:**

Isolated hypoganglionosis is a rare cause of intestinal dysganglionosis and cannot be differentiated from Hirschsprung's disease based on clinical presentation. This case report describes an atypical presentation of the disease. A definitive diagnosis requires histopathological analysis of full-thickness intestinal biopsies. Treatment should be tailored to the extent of hypoganglionosis.

## Introduction

Hypoganglionosis is a hypogenetic variant of intestinal dysganglionosis, characterized by sparse and small myenteric ganglia, absent or low acetylcholinesterase (AchE) activity in the lamina propria and hypertrophy of the muscularis mucosae, principally in the region of the colon and rectum [1]. Hypoganglionosis occurs in two subtypes: the isolated form, and hypoganglionosis associated with Hirschsprung disease [2]. Isolated disease is rare, and accounts for 5% of all the intestinal neuronal malformations [1]. Although such developmental abnormalities as a cause of visceral neuropathy are usually symptomatic and diagnosed in infancy or childhood, we report a case of isolated hypoganglionosis in a 34-year-old woman.

## Case presentation

A 34-year-old Asian woman was admitted to our emergency department with complaints of progressively increasing abdominal pain. She had been unable to pass stool or flatus for two days. Our patient also had a history of chronic constipation that was attributed to irritable bowel syndrome, and had been treated with laxatives and prokinetics without lasting success. She was also reported to have pseudodextrocardia due to elevation of her left hemidiaphragm by dilated loops of bowel. Her medical history and family history were non-contributory.

**Figure 1:**
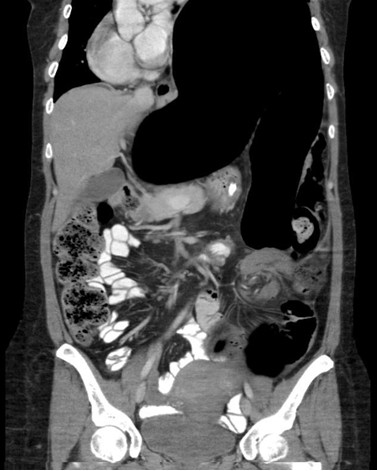
**Computerized tomography scan of the abdomen showing sigmoid volvulus**.

A physical examination was significant for a distended abdomen with sluggish gut sounds. A digital rectal examination was normal. Initial laboratory test results were also within normal limits. Abdominal radiography demonstrated a massively distended sigmoid colon and rectum with dissipated faeces, giving a ground-glass appearance. There was no evidence of pneumoperitoneum. A computerized tomography (CT) scan demonstrated gross dilatation of the sigmoid colon (maximal diameter 14.3 cm) with swirling of mesenteric vessels in the left iliac fossa suggestive of sigmoid volvulus (Figure 1).

**Figure 2:**
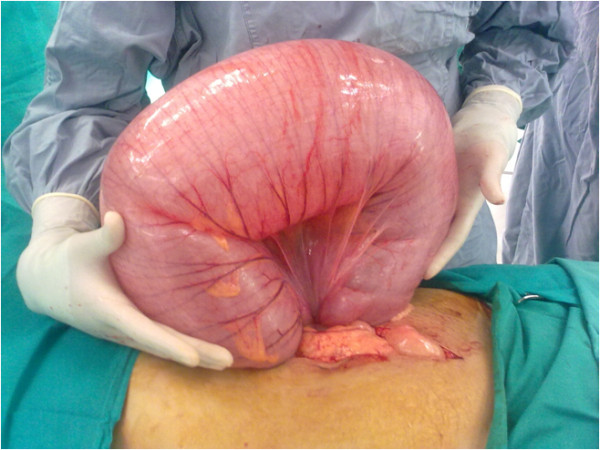
**Operative findings showing large sigmoid volvulus**.

During an emergency laparotomy procedure, we observed megacolon with concurrent sigmoid volvulus with a twist at the mid-descending colon level. Figure 2 shows the large sigmoid volvulus peri-operatively. The bowel was found to be viable and without contamination. After resection of the sigmoid colon, a side-to-side colorectal anastomosis was performed followed by an ileostomy 30 to 40 cm from the ileocolic junction. The sigmoidectomy sample was sent for histopathology analysis.

**Figure 3:**
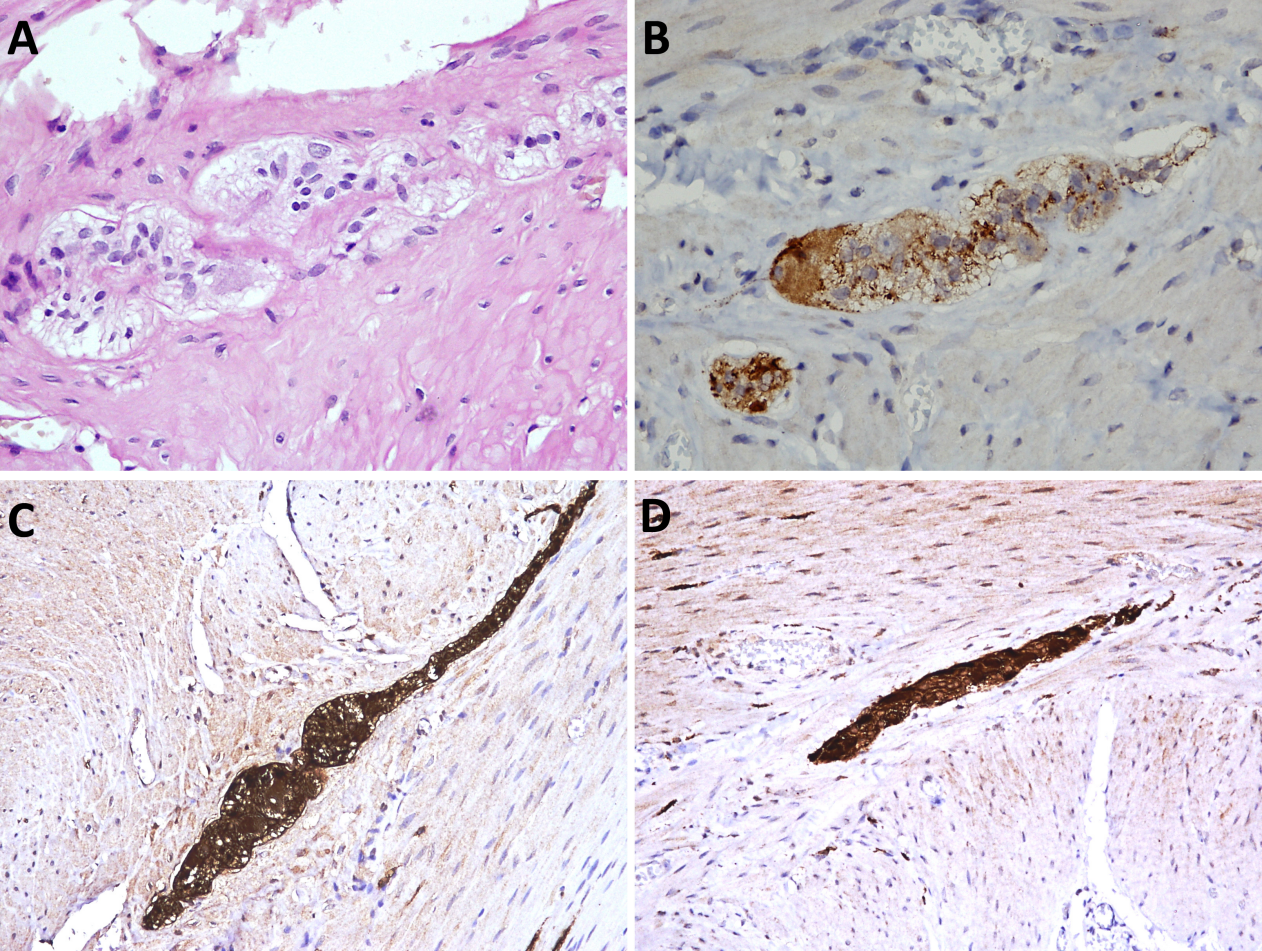
**Immunohistochemical staining of biopsy showing hypertrophied nerve bundles and occasional ganglion cells**. (A) Hematoxylin and eosin stain; (B) calretinin stain; (C) S100 stain; (D) neurofilament stain.

A post-operative CT scan showed no evidence of leakage and our patient remained well. The histopathology examination of sigmoid colon showed occasional ganglion cells and hypertrophied nerve bundles in the muscle layers, suggesting hypoganglionosis. Our patient underwent colonoscopy with biopsy to determine the extent of hypoganglionosis. The colonoscopy showed a dilated bowel with non-obstructing bezoars in the rectum and ascending colon and an anastomotic stricture 25 cm from the anal verge. Full-thickness biopsies were taken from the descending, transverse and ascending colon. The biopsy specimens showed hypoganglionosis in all parts of colon (Figure 3).

**Figure 4:**
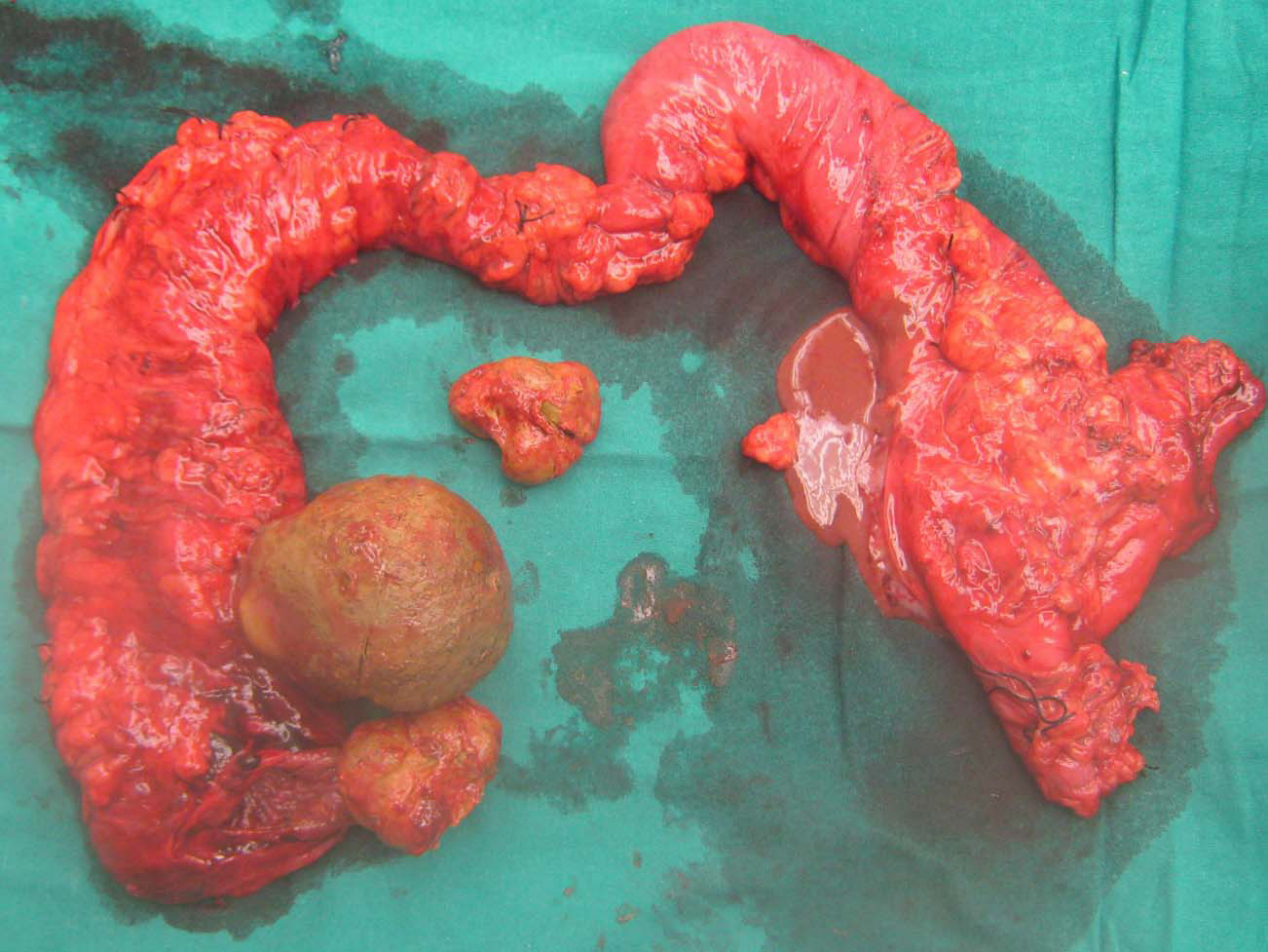
**Resected colon showing two large bezoars**.

Our patient underwent total colectomy with an ileorectal anastomosis followed by reversal of the ileostomy. Figure 4 shows her resected colon with two large bezoars. Our patient has been followed-up for over a year and has reported a dramatic improvement in her bowel function and quality of life.

## Discussion

Hypoganglionosis is a controversial condition because (1) there is no definitive diagnostic criterion for hypoganglionosis based on the location of pathology or the clinical course; (2) hypoganglionosis occurs in two subtypes, isolated hypoganglionosis (IH) and hypoganglionosis associated with Hirschsprung disease (HD) and intestinal neuronal dysplasia; and (3) finally, hypoganglionosis also normally exists immediately above the dentate line. Regardless, isolated hypoganglionosis should be regarded as a distinct condition in which there is decreased number of ganglion cells in the myenteric plexus whereas the submuscosal plexus is normal. However, HD affects both submucosal and myenteric plexuses and the transitional segment (between aganglionic and normal segments of intestine) is called hypoganglionosis [2].

Due to the rarity of the condition, the number of reported cases in the literature is limited. To the best of our knowledge only 92 cases were reported from 1978 to 2009 (69 men and 23 women) [1]. Table 1 gives a review of the literature on isolated hypoganglionosis from 1978 to 2009.

**Table 1:** Review of literature on isolated hypoganglionosis from 1978 to 2009

First author/reference	No. of cases	Specimen	Staining	Treatment	Mortality
Zhang *et al. *[5]	17	Rectal suction and full-thickness biopsy	AchE	Resection and pull through	No
Rolle *et al. *[6]	6	Rectal suction and full-thickness biopsy	AchE, NADPH, ICC, c-kit	5 Resection and pull through, 1 enterostomy	No
Kobayashi *et al. *[2]	3	Full-thickness biopsy	AchE	Enterostomy	All died
Kubota *et al. *[7]	6	Full-thickness biopsy	S-100	Enterostomy	All died
Meier-Ruge *et al. *[3]	7	Full-thickness biopsy	AchE, LDH, SDH	Resection and pull through	No
Schaerli *et al. *[8]	7	Full-thickness biopsy	AchE, LDH, SDH	Resection and pull through	No
Ure *et al. *[9]	9	Rectal suction biopsy	AchE, LDH, SDH	7 resection and pull through, 2 sphincter myotomy	1 died
Yamataka *et al. *[10]	1	Full-thickness biopsy	AchE, ICC, c-kit	Enterostomy	No
Munakata *et al. *[11]	12	Rectal suction and full-thickness biopsy	AchE, silver impregnation	11 resection and pull through, 1 not reported	No
Meier-Ruge [12]	18	Full-thickness biopsy	AchE, LDH, SDH	Not reported	No
Munakata *et al. *[13]	6	Full-thickness biopsy	AchE, silver impregnation	Not reported	No

Adapted from Dingemann J, Puri P: **Isolated hypoganglionosis: systematic review of a rare intestinal innervation defect**. *Pediatr Surg Int ***26**:1111-1115 [1] (Permission granted).

AchE = acetylcholinesterase; ICC = interstitial cells of Cajal; LDH = lactate dehydrogenase; NADPH = nicotinamide adenine dinucleotide phosphate; SDH = succinate dehydrogenase.

The clinical and epidemiological features of patients with IH resemble Hirschsprung's disease, although the median age at diagnosis is significantly higher; that is, 4.85 years (range 3.3 days to 17 years). Our patient was diagnosed at 34 years of age.

Patients usually present with severe acute and chronic constipation, pseudo-obstruction or enterocolitis [1]. In our patient's case, she presented with sigmoid volvulus. To date there has been no report of isolated hypoganglionosis associated with sigmoid volvulus.

According to the literature, a diagnosis of hypoganglionosis can only be established by histopathological staining of full-thickness bowel specimens. Immunohistochemical staining of bowel specimens for acetylcholinesterase, showing low or absent activity of AchE, is used to confirm the diagnosis. This method has been reported as a diagnostic modality in 10 out of 11 reports describing IH from 1978 to 2009 [1]. Morphometric measurements in IH using AchE staining, performed by Meier-Ruge *et al*., represents one of the cornerstones of the diagnostic criteria for the disease. Using resected bowel specimens from patients with hypoganglionosis, they found that the number of nerve cells was only about 40% that of a normal innervated colon and the distance between the ganglia were doubled (hypoganglionosis: 421 ± 98 μm; normal: 174 ± 60 μm). The mean area of the ganglia in hypoganglionosis was three times smaller than the normal innervated colon (hypoganglionosis: 8.48 ± 2.40 mm^2^; normal: 21.88 ± 5.12 mm^2^) [3].

Other commonly used additional markers include staining of the biopsy for lactate dehydrogenase (LDH), succinate dehydrogenase (SDH), nicotinamide adenine dinucleotide phosphate (NADPH)-diaphorase, c-kit, interstitial cells of Cajal (ICC) or silver staining and S-100 staining [1].

In our patient, the presence of concurrent sigmoid volvulus necessitated urgent treatment with emergency laparotomy and sigmoidectomy, whereas diagnosis was made later by histopathological analysis of resected bowel specimens. Beside standard hematoxylin and eosin stain, immunohistochemical stains such as calretinin, neurofilament and S100 were used to identify the ganglion cells and nerve bundles. Immunohistochemical evaluation of acetylcholinesterase, which is the most frequently described staining method for IH in the literature, can only be performed on frozen sections and was therefore not used in this case.

According to the literature, surgery is the definitive treatment method for adult hypoganglionosis and adult Hirschsprung's disease [1]. The principles of pull-through surgery are first to remove all the hypoganglionic segments and second to achieve bowel continuity between the normally innervated bowel and the anal canal in order to provide bowel continence in the long term. The surgical procedures developed to treat the disease in children have been applied to adults without significant differences [4].

The four most commonly used procedures for pull-through surgery are the rectosigmoidectomy developed by Swenson and Bill, the retrorectal approach developed by Duhamel, endorectal procedure developed by Soave and deep anterior colorectal anastomosis developed by Reuben. Of the 67 patients with reported operative procedures, 54 underwent resection/pull-through surgery. Enterostomy was employed as treatment strategy in 11 patients and sphincter myotomy in two patients.

During a post-operative follow-up of seven months to 12 years, typical complications such as enterocolitis, chronic constipation, overflow encopresis, and the need for repeat pull-through surgery for residual disease have been reported. In the 92 cases of isolated hypoganglionosis reported between 1978 and 2009, the overall mortality was 8%. Six of the seven patients that died were newborns suffering from severe enterocolitis; the other patient died owing to total parenteral nutrition-associated complications during follow-up. Our patient has been observed for over a year without any complication.

## Conclusions

Isolated hypoganglionosis is a rare disease with clinical and epidemiological features similar to Hirschsprung's disease, although the age at diagnosis is higher. Careful examination of full-thickness biopsies is required to make a definitive diagnosis.

## Consent

Written informed consent was obtained from the patient for publication of this case report and any accompanying images. A copy of the written consent is available for review by the Editor-in-Chief of this journal.

## Competing interests

The authors declare that they have no competing interests.

## Authors' contributions

IQ and MMS were involved in data collection, literature review and manuscript preparation. AB selected and annotated appropriate images from histopathology slides and reviewed all available histology to ensure an accurate diagnosis was made. HZ was involved in patient care and revised and corrected the manuscript. All authors have read and approved the final manuscript.
